# LigaSure hemorrhoidectomy versus the procedure for prolapse and hemorrhoids: A meta-analysis of randomized controlled trials

**DOI:** 10.1097/MD.0000000000028514

**Published:** 2022-01-21

**Authors:** Leichang Zhang, Yufang Xie, Derong Huang, Xiaofei Ma, Wanchun Wang, Huirong Xiao, Wu Zhong

**Affiliations:** a Department of Anorectal Surgery, The Affiliated Hospital of Jiangxi University of Traditional Chinese Medicine, Nanchang, P.R. China; b Modern Educational Technology Center, Jiangxi Science and Technology Normal University, Nanchang, P.R. China; c Department of Surgery and Traditional Chinese Medicine, The Affiliated Hospital of Jiangxi University of Traditional Chinese Medicine, Nanchang, P.R. China.

**Keywords:** hemorrhoids, LigaSure, meta-analysis, PPH

## Abstract

**Background:**

LigaSure hemorrhoidectomy and the procedure for prolapse and hemorrhoids (PPH) are both relatively new treatments for managing symptomatic hemorrhoids. This review aimed to evaluate and compare their short-term outcomes.

**Methods:**

We searched MEDLINE, EMBASE, the Cochrane Central Register of Controlled Trials and the China National Knowledge Infrastructure database for randomized controlled trials comparing the LigaSure procedure and PPH published in any language from 1998 to October 2013.

**Results:**

A total of 5 studies involving 397 participants were included in this review. Pooled analysis showed that the LigaSure procedure was associated with significantly lower recurrence rate [relative risk (RR) = 0.21, 95% confidence interval (CI): 0.06 to 0.72, *P* = .01] and significantly shorter operating time [mean difference (MD) = −6.39, 95% CI: −7.68 to −5.10, *P* < .001]. The analysis showed no significant difference in postoperative pain between the two techniques (MD = 0.55, 95% CI: −0.15 to 1.25, *P* = .12] or in time off work or away from normal activity [standard MD = 0.13, 95% CI: −1.80 to 2.06, *P* = .9]. The two techniques did not show significant differences in postoperative complications or other patient-related outcomes (*P* > .05).

**Conclusions:**

Our review indicates that both LigaSure hemorrhoidectomy and PPH are safe alternatives for the management of hemorrhoids. Available evidence suggests that the LigaSure technique is associated with shorter operating time and lower hemorrhoid recurrence rate, but these conclusions should be further confirmed in large, multicenter randomized controlled trials with long-term follow-up.

## 1 Introduction

Hemorrhoids are a physiological component of the anal canal consisting mainly of vascular tissue and supported by smooth muscle and connective tissue.^[1]^ The hemorrhoidal cushions, which provide additional compression to close the anus completely, are found predominantly at three positions of the anal canal: left lateral (3 o’clock), right anterolateral (7 o’clock) and right posterolateral (11 o’clock).^[2]^ Hemorrhoid disease, a term that usually refers to hypertrophy of the hemorrhoidal plexus and pathological changes in the anal cushions,^[3]^ is one of the most common anorectal disorders, with a prevalence of 4.4% to 86% according to various studies.^[4–7]^ The age distribution of the disease shows a Gaussian distribution with a peak incidence between 45 and 65 years.^[8]^ The prevalence may be underestimated since only about one-third of patients with the disease turn to physicians for advice.^[8,9]^ Though men are more likely to seek treatment than women, the incidence of the disease is similar in both genders.^[10]^ Hemorrhoid disease invariably results in such symptoms as rectal bleeding, painful defecation, inflammation, mucosal prolapse or protrusion, and pruritis ani.^[1]^ Risk factors for the disease include a low-fiber diet, prolonged straining, constipation, diarrhea and hard stool.^[11]^

Hemorrhoidectomy is recommended as the definitive treatment for grade III and IV hemorrhoidal disease.^[12]^ Indeed, most surgeons rely on hemorrhoidectomy to manage symptomatic hemorrhoids. Both open^[13]^ and closed^[14]^ techniques are widely used, and both are associated with similar postoperative pain outcomes and complications.^[15,16]^ Postoperative pain is usually substantial, and complications include urinary retention, bleeding and subcutaneous abscess anal fissure, anal stenosis, incontinence, fistula, and hemorrhoid recurrence.^[17,18]^ As a result, significant efforts have been made to develop new treatment approaches.

In 1998, Longo^[19]^ introduced a novel procedure referred to as stapled hemorrhoidectomy, hemorrhoidopexy, or the procedure for prolapse and hemorrhoids (PPH). Several early randomized controlled trials (RCTs) showed PPH to be less painful than traditional excisional surgery.^[20–27]^ More recently, the LigaSure vessel sealing system, designed initially for abdominal surgery, has proven effective at reducing post-hemorrhoidectomy pain significantly below that of conventional hemorrhoidectomy.^[28–30]^ The LigaSure technique allows complete coagulation of vessels up to 7 mm in diameter such that thermal spread and tissue charring are minimized.^[31,32]^ In fact, the technique restricts thermal spread to within 2 mm of the adjacent tissue, helping to minimize anal spasm and pain.^[29,30]^

LigaSure and PPH are relatively new procedures and we are unaware of systematic attempts to compare their safety and efficacy. Such comparisons may prove invaluable for helping clinicians decide which approach to use when managing symptomatic hemorrhoids. Therefore we carried out a meta-analysis of RCTs analyzing short-term outcomes of the two treatments.

## 2 Materials and method

### 2.1 Search strategy

This meta-analysis does not require an ethics approval as it does not collect any primary data from patients. We searched MEDLINE, EMBASE, the Cochrane Central Register of Controlled Trials and the China National Knowledge Infrastructure databases without language restrictions from 1998, the year when Longo^[19]^ first reported stapled hemorrhoidectomy, until October 2013. We used the following search terms: hemorrhoid, haemorrhoid, pile, PPH, longo, stapl^∗^, random^∗^, and Ligasure. The reference lists in relevant studies were manually searched to identify additional potential studies. We also searched in the clinical trial registries at ClinicalTrials.Gov (www.clinicaltrials.gov) and in the Meta Register of Controlled Trials (mRCT) (www.controlled-trials.com).

### 2.2 Inclusion and exclusion criteria

Only prospective RCTs comparing LigaSure and PPH for treating hemorrhoids were eligible for the review. All patients with symptomatic hemorrhoids who underwent either procedure were eligible for inclusion, irrespective of age, gender or hemorrhoid grade. Patients who underwent hemorrhoidectomy with the LigaSure vessel sealing device were assigned to the LigaSure group. Patients who underwent hemorrhoidectomy with a custom-designed stapler were assigned to the PPH group. Retrospective and non-randomized comparative studies, cohort studies, case series and case reports were excluded. When two or more studies showed substantial overlap of participants, study duration, authors or institutions, we included only the most recent or highest-quality study.

### 2.3 Study selection and data extraction

Two reviewers independently performed primary screening of potentially eligible studies based on the title, abstract and MeSH terms. All articles selected through primary screening were then read in full to ensure study eligibility. Any disagreements were resolved by discussion with a third reviewer. Data on baseline information and outcomes were extracted from the final set of included studies using specially designed tables. If studies were found to report insufficient information, attempts were made to contact authors.

### 2.4 Outcome measures

Primary outcomes were postoperative pain, recurrence rate, and time off work or away from normal activity. Secondary outcomes included postoperative complications (hemorrhage, urinary retention, stenosis, itching, difficult defecation, and incontinence), and other patient-related results (hospital stay, need for analgesics, and operating time).

### 2.5 Quality assessment

The quality of included RCTs was assessed using the five-point Jadad scale.^[33]^ Studies with a score of less than three were regarded as low quality, while trials with a score of three or more were regarded as high quality. We used GradePro 3.6^[34]^ designed by the Cochrane Collaboration to further evaluate the strength of evidence for primary outcomes.

### 2.6 Patient and public involvement

Patients and the public were not involved in this meta-analysis.

### 2.7 Statistical methods

All statistical analyses were performed using Review Manager 5.1, designed by the Cochrane Collaboration. Pooled dichotomous data were analyzed using the risk ratio (RR) with 95% confidence intervals (CIs). Pooled continuous data were analyzed using the mean difference (MD) if outcomes were measured in the same way among trials, or the standard mean difference (SMD) if outcomes were reported in different units. Heterogeneity among trials in each analysis was assessed using the *I*^2^ statistic.^[35]^ We defined heterogeneity as substantial when *I*^2^ was more than 50%, in which case pooled data were analyzed using a random effect model (REM); otherwise, if *I*^2^ was less than 50%, data were analyzed using a fixed effect model (FEM).

### 2.8 Sensitive analysis

The robustness of results was tested by sensitivity analyses in which the outcomes from the REM and FEM were compared; robust results should not be affected by changing the model. Outcomes were presented descriptively when meta-analysis could not be carried out.

## 3 Results

### 3.1 Characteristics of included studies

**Figure 1:**
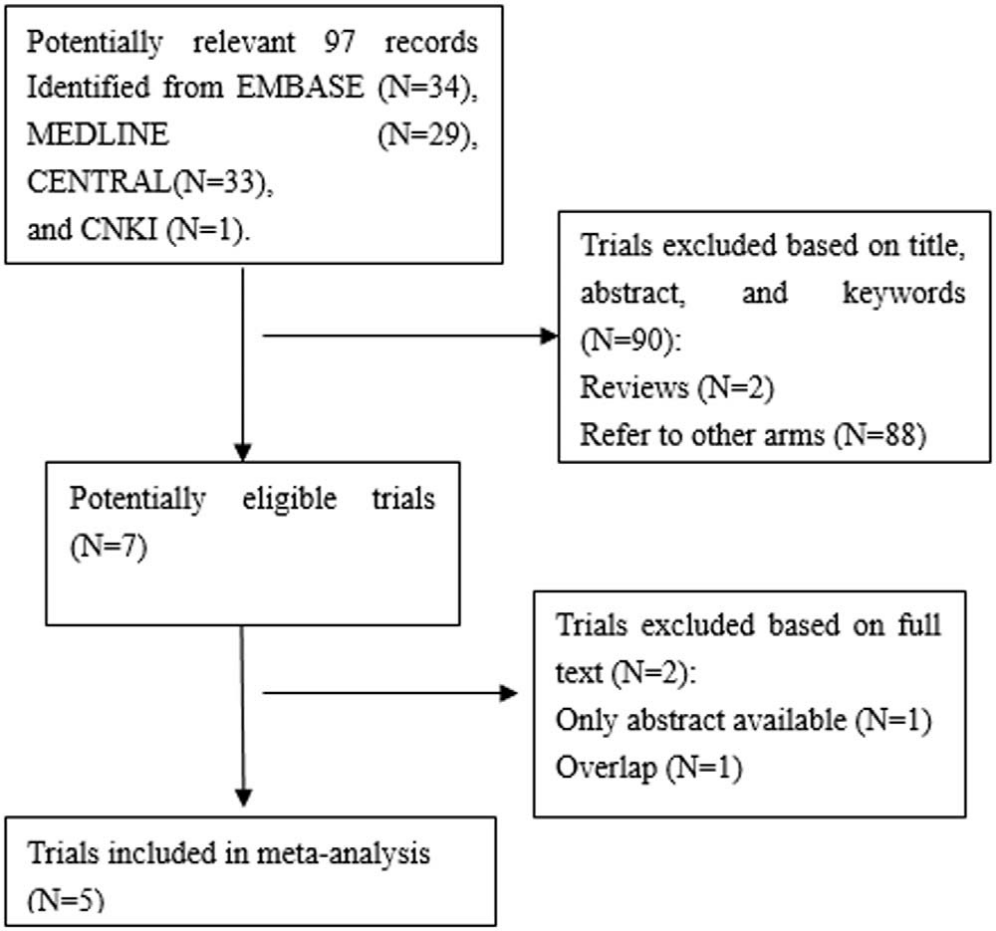
Flow chart of study selection.

Our literature search identified 97 potentially relevant studies (Fig. 1). Most studies could be eliminated during the primary screening of titles, abstracts and keywords. The full text of the remaining 7 trials was read. One study was excluded because only the abstract was available,^[36]^ while another was excluded because of overlap.^[37]^ Finally, 5 studies^[38–42]^ involving 397 participants were found to be eligible for this review. The sample size of included studies ranged from 50 to 98. Demographic data were reported in all included studies and were statistically comparable (Table 1).

**Table 1:** Key characteristics of studies included in the systematic review.

Study	Country	LigaSure/PPH (N)	Male:female (N), LigaSure/PPH	Age, yr^∗^	Hemorrhoid grade	Follow up	Jadad score
Arslani 2012^[40]^	Croatia	52/46	23:29, 21:25	50 (18–78)52 (17–72)	III	24 mo.	3
Sakr 2010^[41]^	Egypt	34/34	19:15, 21:13	39.3352 (17–72)	III, IV	18 mo.	4
Chen 2007^[42]^	China	42/44	24:18, 26:18	46 (23–85)48 (25–81)	III	6 mo.	3
Kraemer 2005^[38]^	Germany	25/25	13:12, 14:11	58 (28–72)58 (40–71)	III, IV	6 wk	3
Basdanis 2005^[39]^	Greece	45/50	NR	NR	III, IV	24 mo.	3

### 3.2 Quality assessment of included studies

All the included studies were single-center RCTs with moderate Jadad scores. All studies had scores of 3, except one study^[41]^ with a score of 4 because it reported single blinding. We assessed the strength of evidence about primary outcomes using GradePro. The strength of evidence about recurrence rate was high; about postoperative pain, low; and about time off work, very low.

### 3.3 Sensitive analysis

We performed the sensitive analysis of all the pooling results by changing the effect model, and all the findings were stabilized and shown under the appropriate effect model.

### 3.4 Postoperative pain

**Figure 2:**
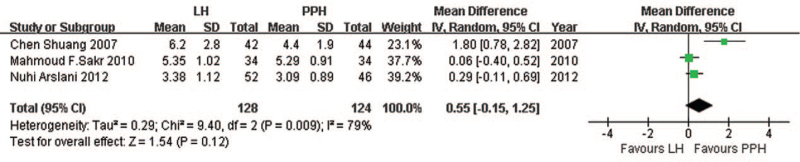
Comparison of postoperative pain following LigaSure hemorrhoidectomy or PPH. PPH = procedure of prolapse and hemorrhoids.

Three studies^[40–42]^ reported mean results for postoperative pain and found no significant difference between LigaSure and PPH (MD = 0.55, 95% CI: −0.15 to 1.25, *P* = .12, REM; heterogeneity, *I*^2^ = 79%, *P* = .009; Fig. 2). Another study^[38]^ reporting mean results (without data of standard deviation) on postoperative pain came to a similar conclusion. One study^[39]^ reported postoperative pain results but it could not be included in the pooled analysis because it reported outcomes as medians and ranges. Median visual analogue scale (VAS) scores (range) were significantly lower for PPH than for LigaSure at various follow-up points: after 8 hours, 3 (2–6) vs 5 (3–8), *P* < .01; after 24 hours, 3 (1–6) vs 6 (3–7), *P* < .01; after the first defecation, 5 (3–8) vs 7 (3–9), *P* < .001.

### 3.5 Hemorrhoid recurrence rate

**Figure 3:**
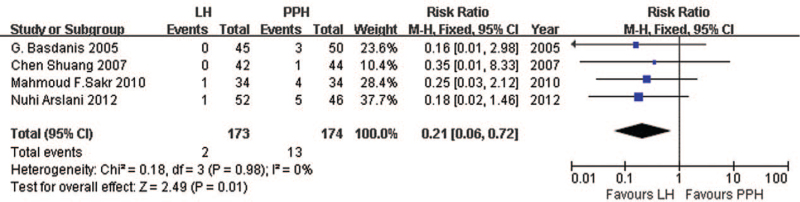
Comparison of hemorrhoid recurrence rate following LigaSure hemorrhoidectomy or PPH. PPH = procedure of prolapse and hemorrhoids.

Four studies^[39–42]^ reported data on hemorrhoid recurrence. Pooled analysis showed that the recurrence rate was significantly lower for LigaSure than for PPH (RR = 0.21, 95% CI: 0.06 to 0.72, *P* = .01, FEM; heterogeneity, *I*^2^ = 0%, *P* = .98; Fig. 3).

### 3.6 Time off work or away from normal activity

**Figure 4:**
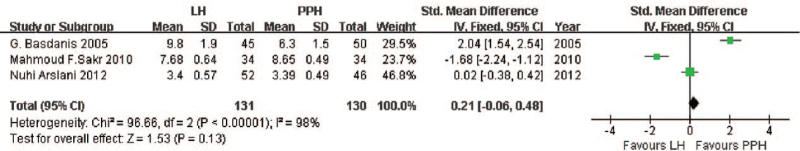
Comparison of time off work or away from normal activity following LigaSure hemorrhoidectomy or PPH. PPH = procedure of prolapse and hemorrhoids.

Three studies reported data about time off work or away from normal activity.^[39–41]^ This outcome did not differ significantly between the two techniques (SMD = 0.13, 95% CI: −1.80 to 2.06, *P* = .9, REM; heterogeneity, *I*^2^ = 98%, *P* < .001; Fig. 4).

### 3.7 Postoperative complications

**Figure 5:**
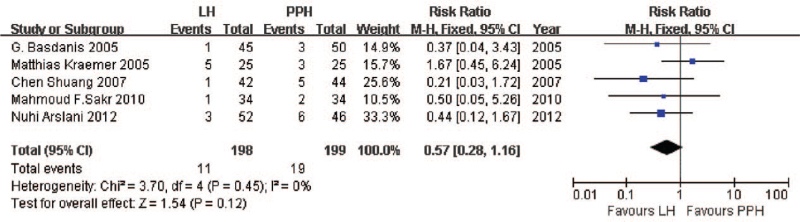
Comparison of hemorrhage occurrence following LigaSure hemorrhoidectomy or PPH. PPH = procedure of prolapse and hemorrhoids.

**Figure 6:**
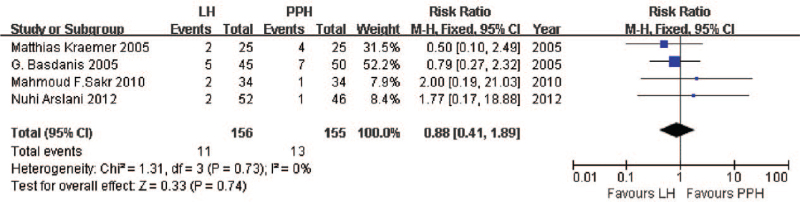
Comparison of urinary retention following LigaSure hemorrhoidectomy or PPH. PPH = procedure of prolapse and hemorrhoids.

While not all the included studies reported data on the same postoperative complications, the available data showed no significant differences between LigaSure and PPH. All the included studies reported data about hemorrhaging, pooled analysis indicated no significant difference between the two techniques (RR = 0.57, 95% CI: 0.28 to 1.16, *P* = .12, FEM; heterogeneity, *I*^2^ = 0%, *P* = .45; Fig. 5). Pooled analysis of urinary retention^[39–42]^ showed no significant difference (RR = 0.88, 95% CI: 0.41 to 1.89, *P* = .74, FEM; heterogeneity, *I*^2^ = 0%, *P* = .73; Fig. 6). Similar results were obtained for stenosis^[38,40–41]^ (RR = 0.80, 95% CI: 0.20 to 3.17, *P* = .75, FEM; heterogeneity, *I*^2^ = 0%, *P* = .63), itching^[38–39,42]^ (RR = 1.58, 95% CI: 0.32 to 7.79, *P* = .57, REM; heterogeneity, *I*^2^ = 86%, *P* < .001), difficult defecation^[38–39,41–42]^ (RR = 0.89, 95% CI: 0.46 to 1.69, *P* = .71, FEM; heterogeneity, *I*^2^ = 0%, *P* = .58), and incontinence^[38–41]^ (RR = 0.50, 95% CI: 0.15 to 1.60, *P* = .24, FEM; heterogeneity, *I*^2^ = 0%, *P* = .99).

### 3.8 Other patient-related outcomes

Only two studies^[41–42]^ reported mean hospital stays; pooled analysis showed no significant difference between LigaSure and PPH (RR = 0.82, 95% CI: −1.27 to 2.91, *P* = .44, REM; heterogeneity, *I*^2^ = 98%, *P* < .001). Similar results were obtained for the rate of patients needing analgesics in three studies^[38,40,42]^ (RR = 1.06, 95% CI: 0.81 to 1.40, *P* = .65, REM; heterogeneity, *I*^2^ = 88%, *P* < .001). In contrast, pooled analysis of operating time^[41,42]^ showed a significantly shorter time for the LigaSure procedure (MD = −6.39, 95% CI: −7.68 to −5.10, *P* < .001, FEM; heterogeneity, *I*^2^ = 0%, *P* = .52).

## 4 Discussion

This is, to our knowledge, the first meta-analysis comparing the safety and efficacy of LigaSure hemorrhoidectomy and PPH, two relatively new approaches to managing symptomatic hemorrhoids. Our meta-analysis shows that the LigaSure technique is associated with significantly lower hemorrhoid recurrence and shorter operating time. The two techniques are similar in terms of postoperative pain, time off work, hemorrhage, urinary retention, stenosis, itching, difficult defecation, incontinence, hospital stay, and the need for analgesics.

Therapies for hemorrhoid disease fall into three main categories:

(1) conservative therapy, which seeks to alter bowel habits and ensure sufficient dietary fiber intake^[43]^;
(2) medical therapy, including such techniques as sclerotherapy, rubber band ligation, cryotherapy, infrared coagulation, laser therapy and diathermy coagulation^[44]^; and
(3) surgical therapy, involving such techniques as conventional hemorrhoidectomy (open or closed), LigaSure hemorrhoidectomy, PPH, and Doppler-guided transanal hemorrhoidal de-arterialization. Hemorrhoidectomy is usually the final and effective choice for treating symptomatic hemorrhoids, but the conventional procedure is associated with substantial postoperative pain and various complications.

All but one of the included studies were of moderate quality, with a Jadad score of 3. One study^[41]^ incorporated single blinding and therefore had a score of 4. All studies reported appropriate randomization methods, although none described allocation concealment. The strength of evidence about recurrence rate was evaluated as strong, suggesting that it should be taken into serious consideration during clinical decision-making. In contrast, the strength of evidence was low for results about postoperative pain and very low for results about time off work or away from normal activity.

The complications reported in the studies in our review were not so serious as those previously reported for PPH, which include pelvic sepsis,^[45–47]^ rectal obstruction,^[48]^ rectal perforation,^[49–50]^ rectovaginal fistula^[45]^, and staple line dehiscence.^[51]^ This may reflect PPH is relatively safe provided that we restrictively followed the operative indication and completed the operation with experienced skills. A Cochrane systematic review^[52]^ comparing PPH and conventional hemorrhoidectomy showed PPH to be associated with a higher long-term risk of hemorrhoid recurrence and similar postoperative pain. Our systematic review extends these findings by showing that PPH is also associated with a higher recurrence rate and similar postoperative pain as the LigaSure approach.

This systematic review suffers from several limitations. First, it is based on a small number of studies with relatively small samples. Second, most studies were not of high quality, with only one study describing any blinding and none of the studies describing allocation concealment. Third, incomplete reporting in the included studies prevented us from performing subgroup analyses based on hemorrhoid grade or length of follow-up. Fourth, we included only studies for which the full text was available, which may have introduced selection bias.

Our systematic review of the literature comparing LigaSure hemorrhoidectomy and PPH leads us to recommend that future RCTs be large, multi-centered, and double-blinded; that they include long-term follow-up and perform subgroup comparisons based on hemorrhoid grade; and that they take into account additional outcomes such as cost-effectiveness and postoperative quality of life.

## 5 Conclusion

Our review indicates that both LigaSure hemorrhoidectomy and PPH are safe options for treating hemorrhoids. Available evidence suggests that the LigaSure technique is associated with a lower recurrence rate and operating time, but these findings should be validated in larger, multi-center RCTs involving long-term follow-up.

## Acknowledgments

The authors thank Dr Yun-Fei Cao and Dr Chapin for their language editing and other suggestions, which helped improve the manuscript.

## Author contributions

**Conceptualization:** Leichang Zhang, Yufang Xie, Wu Zhong.

**Data curation:** Leichang Zhang, Yufang Xie, Wu Zhong.

**Formal analysis:** Leichang Zhang, Yufang Xie.

**Methodology:** Derong Huang.

**Resources:** Xiaofei Ma.

**Software:** Xiaofei Ma.

**Validation:** Wanchun Wang.

**Visualization:** Wanchun Wang.

**Writing – original draft:** Wu Zhong.

**Writing – review & editing:** Huirong Xiao, Wu Zhong.

## References

[1] Thomson WHF. The nature of haemorrhoids. Br J Surg 1975;62:542–52. 1174785 10.1002/bjs.1800620710

[2] Brisinda G. How to treat haemorrhoids. Prevention is best; haemorrhoidectomy needs skilled operators. Br Med J 2000;321:582–3. 10977817 10.1136/bmj.321.7261.582 PMC1118483

[3] Nisar PJ Scholefield JH. Managing haemorrhoids. Br Med J 2003;327:847–51. 14551102 10.1136/bmj.327.7419.847 PMC214027

[4] Dal Monte PP Tagariello C Sarago M. Transanal haemorrhoidal dearterialisation: nonexcisional surgery for the treatment of haemorrhoidal disease. Tech Coloproctol 2007;11:333–8. 18060529 10.1007/s10151-007-0376-4

[5] Carapeti EA Kamm MA McDonald PJ. Double-blind randomised controlled trial of effect of metronidazole on pain after day-case haemorrhoidectomy. Lancet 1998;351:169–72. 9449871 10.1016/S0140-6736(97)09003-X

[6] Jayaraman S Colquhoun PH Malthaner RA. Stapled hemorrhoidopexy is associated with a higher long-term recurrence rate of internal hemorrhoids compared with conventional excisional hemorrhoid surgery. Dis Colon Rectum 2007;50:1297–305. 17665254 10.1007/s10350-007-0308-4

[7] Walega P Scheyer M Kenig J. Two-center experience in the treatment of hemorrhoidal disease using Doppler-guided hemorrhoidal artery ligation: functional results after 1-year follow-up. Surg Endosc 2008;22:2379–83. 18622559 10.1007/s00464-008-0030-x

[8] Johanson JF Sonnenberg A. The prevalence of haemorrhoids andchronic constipation:an epidemiological study. Gastroenterology 1990;98:380–6. 2295392 10.1016/0016-5085(90)90828-o

[9] Nelson RL Abcarian H Davis FG. Prevalence of benign anorectal disease in a randomly selected population. Dis Colon Rectum 1995;38:341–4. 7720437 10.1007/BF02054218

[10] Hussain JN. Hemorrhoids. Primary Care 1999;26:35–51. 9922293 10.1016/s0095-4543(05)70100-7

[11] Haas PA Fox TAJR Haas GP. The pathogenesis of haemorrhoids. Dis Colon Rectum 1984;22:442–50. 10.1007/BF02555533 6745015

[12] Cataldo P Ellis CN Gregorcyk S. Practice parameters for the management of hemorrhoids (revised). Dis Colon Rectum 2005;48:189–94. 15711856 10.1007/s10350-004-0921-4

[13] Milligan ET Morgan CN Jones LE. Surgical anatomy of the anal canal and the operative treatment of haemorrhoids. Lancet 1937;2:1119–24.

[14] Ferguson JA Mazier WP Ganchrow MI. The closed technique of hemorrhoidectomy. Surgery 1971;70:480–4. 5568533

[15] Arbman G Krook H Haapaniemi S. Closed vs. open hemorrhoidectomy: is there any difference? Dis Colon Rectum 2000;43:31–4. 10813120 10.1007/BF02237240

[16] Gencosmanoglu R Sad O Koc D. Hemorrhoidectomy: open or closed technique? A prospective, randomized clinical trial. Dis Colon Rectum 2002;45:70–5. 11786767 10.1007/s10350-004-6116-1

[17] Bleday R Pena JP Rothenberger DA. Symptomatic hemorrhoids: current incidence and complications of operative therapy. Dis Colon Rectum 1992;35:477–81. 1568400 10.1007/BF02049406

[18] Sardinha TC Corman ML. Haemorrhoids. Surg Clin North Am 2002;82:1153–67. 12516845 10.1016/s0039-6109(02)00082-8

[19] Longo A. The pathogenesis of haemorrhoids. Proceedings of the 6th World Congress of Endoscopic Surgery of the 6th World Congress of Endoscopic Surgery 1998; Rome, Italy 777–84.

[20] Ganio E Altomare DF Gabrielli F. Prospective randomized multicentre trial comparing stapled with open haemorrhoidectomy. Br J Surg 2001;88:669–74. 11350437 10.1046/j.0007-1323.2001.01772.x

[21] Hasse C Sitter H Brune M. Haemorrhoidectomy: conventional excision versus resection with the circular stapler. Prospective, randomized study. Deutsche Medizinische Wochenschrift 2004;129:1611–7. 15257499 10.1055/s-2004-829001

[22] Ho YH Cheong WK Tsang C. Stapled hemorrhoidectomy-cost and effectiveness. Randomized, controlled trial including incontinence scoring, anorectal manometry, and endoanal ultrasound assessments at up to three months. Dis Colon Rectum 2000;43:1666–75. 11156449 10.1007/BF02236847

[23] Khalil KH O’Bichere A Sellu D. Randomized clinical trial of sutured versus stapled closed haemorrhoidectomy. Br J Surg 2000;87:1252–355. 10.1046/j.1365-2168.2000.01624.x 11044160

[24] Kirsch JJ Staude G Herold A. The Longo and Milligan-Morgan hemorrhoidectomy. A prospective comparative study of 300 patients. Chirurg 2001;72:180–5. 11253679 10.1007/s001040051289

[25] Rowsell M Bello M Hemingway DM. Circumferential mucosectomy (stapled haemorrhoidectomy) versus conventional haemorrhoidectomy: randomised controlled trial. Lancet 2000;355:779–81. 10711924 10.1016/s0140-6736(99)06122-x

[26] Schmidt MP Fischbein J Shatavi H. Stapler hemorrhoidectomy versus conventional procedures – a clinical study. Zentralbl Chir 2002;127:15–8. 11889632 10.1055/s-2002-20226

[27] Shalaby R Desoky A. Randomized clinical trial of stapled versus Milligan-Morgan haemorrhoidectomy. Br J Surg 2001;88:1049–53. 11488788 10.1046/j.0007-1323.2001.01830.x

[28] Franklin EJ Seetharam S Lowney J. Randomized, clinical trial of Ligasure vs. conventional diathermy in hemorrhoidectomy. Dis Colon Rectum 2003;46:1380–3. 14530679 10.1007/s10350-004-6754-3

[29] Milito G Gargiani M Cortese F. Randomised trial comparing LigaSure haemorrhoidectomy with the diathermy dissection operation. Tech Coloproctol 2002;6:171–5. 12525911 10.1007/s101510200038

[30] Thorbeck CV Montes MF. Haemorrhoidectomy: randomised controlled clinical trial of Ligasure compared with Milligan-Morgan operation. Eur J Surg 2002;168:482–4. 12549689 10.1080/110241502321116497

[31] Kennedy JS Stranahan PL Taylor KD. High-burst-strength, feedback controlled bipolar vessel sealing. Surg Endosc 1998;12:876–8. 9602010 10.1007/s004649900733

[32] Palazzo FF Francis DL Clifton MA. Randomized clinical trial of Ligasure versus open haemorrhoidectomy. Br J Surg 2002;89:154–7. 11856126 10.1046/j.0007-1323.2001.01993.x

[33] Jadad AR Moore RA Carroll D. Assessing the quality of reports of randomized clinical trials: is blinding necessary? Control Clin Trials 1996;17:01–12. 10.1016/0197-2456(95)00134-4 8721797

[34] Cochrane Collaboration. gradepro. Available at: http://ims.cochrane.org/revman/other-resources/gradepro/download. Accessed April 30, 2012.

[35] Higgins JP Thompson SG. Quantifying heterogeneity in a meta-analysis. Stat Med 2002;21:1539–58. 12111919 10.1002/sim.1186

[36] Stolfi VM Sileri P Micossi C. Hemorrhoidectomy in day surgery: a comparison between four techniques. Gastroenterology 2008;134:864.

[37] Sakr MF Moussa MM. LigaSure hemorrhoidectomy versus stapled hemorrhoidopexy: a prospective, randomized clinical trial. Dis Colon Rectum 2010;53:1161–7. 20628280 10.1007/DCR.0b013e3181e1a1e9

[38] Kraemer M Parulava T Roblick M. Prospective, randomized study: proximate (registered trademark) PPH Stapler vs. LigaSure (trademark) for hemorrhoidal surgery. Dis Colon Rectum 2005;48:1517–22. 15937619 10.1007/s10350-005-0067-z

[39] Basdanis G Papadopoulos VN Michalopoulos A. Randomized clinical trial of stapled hemorrhoidectomy vs open with Ligasure for prolapsed piles. Surg Endosc 2005;19:235–9. 15573239 10.1007/s00464-004-9098-0

[40] Arslani N Patrlj L Rajkovic Z. A randomized clinical trial comparing Ligasure versus stapled hemorrhoidectomy. Surg Laparosc Endosc Percut Tech 2012;22:58–61. 10.1097/SLE.0b013e318247d966 22318061

[41] Sakr MF Moussa MM Elserafy M. Ligasure hemorrhoidectomy versus Stapled hemorrhoidopexy: a prospective randomized clinical trial. Minerva Chirurgica 2010;65:251–8. 20668414

[42] Chen S Lai DM Yang B. Therapeutic comparison between procedure for prolapse and hemorrhoids and Ligasure technique for hemorrhoids. Zhonghua wei chang wai ke za zhi = Chinese J Gastrointest Surg 2007;10:342–5. 17659458

[43] Johanson JF. Evidence-based approach to the treatment of hemorrhoidal disease. Evid Based Gastroenterol 2002;3:26–31.

[44] Shanmugam V Thaha MA Rabindranath KS. Rubber band ligation versus excisional haemorrhoidectomy for haemorrhoids. Cochrane Database Syst Rev 2005;CD005034. 10.1002/14651858.CD005034.pub2 PMC8860341 16034963

[45] Lehur PA Gravie JF Meurette G. Circular stapled anopexy for haemorrhoidal disease: results. Colorect Dis 2001;3:374–9. 10.1046/j.1463-1318.2001.00287.x 12790933

[46] Pessaux P Lermite E Tuech JJ. Pelvic sepsis after stapled hemorrhoidectomy. J Am Coll Surg 2004;199:824–5. 15501126 10.1016/j.jamcollsurg.2004.04.026

[47] Molloy RG Kingsmore D. Life threatening pelvic sepsis after stapled haemorrhoidectomy. Lancet 2000;355:810. 10711934 10.1016/S0140-6736(00)02208-X

[48] Cipriani S Pescatori M. Acute rectal obstruction after PPH stapled haemorrhoidectomy. Colorect Dis 2002;4:367–70. 10.1046/j.1463-1318.2002.00409.x 12780584

[49] Ripetti V Caricato M Arullani A. Rectal perforation, retropneumoperitoneum, and pneumomediastinum after stapling procedure for prolapsed hemorrhoids: report of a case and subsequent considerations. Dis Colon Rectum 2002;45:268–70. 11852343 10.1007/s10350-004-6159-3

[50] Wong LY Jiang JK Chang SC. Rectal perforation: a life-threatening complication of stapled hemorrhoidectomy – report of a case. Dis Colon Rectum 2003;46:116–7. 12544531 10.1007/s10350-004-6505-5

[51] Pescatori M. Stapled hemorrhoidectomy: a word of caution. Int J Colorect Dis 2002;17:362–3. 10.1007/s00384-002-0419-2 12420732

[52] Jayaraman S Colquhoun PH Malthaner RA. Stapled versus conventional surgery for hemorrhoids. Cochrane Database Syst Rev 2006;CD005393. 10.1002/14651858.CD005393.pub2 PMC8887551 17054255

